# Evaluation of the quality of care of a multi-disciplinary Risk Factor Assessment and Management Programme for Hypertension (RAMP-HT)

**DOI:** 10.1186/s12875-015-0291-0

**Published:** 2015-06-19

**Authors:** Esther Yee Tak Yu, Eric Yuk Fai Wan, Karina Hiu Yen Chan, Carlos King Ho Wong, Ruby Lai Ping Kwok, Daniel Yee Tak Fong, Cindy Lo Kuen Lam

**Affiliations:** Department of Family Medicine and Primary Care, The University of Hong Kong, Ap Lei Chau, Hong Kong; Primary and Community Services Department, Hospital Authority Head Office, Hong Kong Hospital Authority, Kowloon, Hong Kong; School of Nursing, The University of Hong Kong, Pokfulam, Hong Kong

**Keywords:** Hypertension, Management programme, Multi-disciplinary, Primary Care, Quality of care, Risk Assessment, Risk Stratification

## Abstract

**Background:**

There is some evidence to support a risk-stratified, multi-disciplinary approach to manage patients with hypertension in primary care. The aim of this study is to evaluate the quality of care (QOC) of a multi-disciplinary Risk Assessment and Management Programme for Hypertension (RAMP-HT) for hypertensive patients in busy government-funded primary care clinics in Hong Kong. The objectives are to develop an evidence-based, structured and comprehensive evaluation framework on quality of care, to enhance the QOC of the RAMP-HT through an audit spiral of two evaluation cycles and to determine the effectiveness of the programme in reducing cardiovascular disease (CVD) risk.

**Method/Design:**

A longitudinal study is conducted using the Action Learning and Audit Spiral methodologies to measure whether pre-set target standards of care intended by the RAMP-HT are achieved. A structured evaluation framework on the quality of structure, process and outcomes of care has been developed based on the programme objectives and literature review in collaboration with the programme workgroup and health service providers. Each participating clinic is invited to complete a structure of care evaluation questionnaire in each evaluation cycle. The data of all patients who have enrolled into the RAMP-HT in the pre-defined evaluation periods are used for the evaluation of the process and outcomes of care in each evaluation cycle. For evaluation of the effectiveness of RAMP-HT, the primary outcomes including blood pressure (both systolic and diastolic), low-density lipoprotein cholesterol and estimated 10-year CVD risk of RAMP-HT participants are compared to those of hypertensive patients in usual care without RAMP-HT.

**Discussion:**

The QOC and effectiveness of the RAMP-HT in improving clinical and patient-reported outcomes for patients with hypertension in normal primary care will be determined. Possible areas for quality enhancement and standards of good practice will be established to inform service planning and policy decision making.

**Electronic supplementary material:**

The online version of this article (doi:10.1186/s12875-015-0291-0) contains supplementary material, which is available to authorized users.

## Background

Hypertension (HT) is a global public health issue and an important risk factor for coronary heart diseases, cerebrovascular diseases and renal failure [1, 2]. In Hong Kong, the condition affects 27.2 % of people aged over 15 years and more than 65 % of elderly [3]. Approximately 200,000 people with HT are currently receiving care at government-funded primary care clinics (i.e. General Outpatient Clinics (GOPC) operated by the Hospital Authority (HA)). The majority of these GOPC attendants are elderly. With the threat of aging population and the foreseeable increase in demand of public primary health services to provide chronic disease care, a sustainable and effective management programme to improve the quality of care for HT and to lessen public healthcare burden deems a good solution.

Assessment of total cardiovascular risk has been advocated internationally as a holistic and cost-effective way to guide intervention for patients with HT, where resources can be directed to patients with highest risk in order to achieve maximal prevention or reduction in cardiovascular diseases (CVD) and mortalities [2, 4–6]. The Ontario Medical Association found that comprehensive screening and management via stratification of risk factors in hypertensive patients could double the reduction in cardiovascular risk, lower the blood pressure and change the types of antihypertensive medications recommended [7–9]. The Joint British Societies (JBS) recommended prediction of the total risk of developing cardiovascular diseases (i.e. coronary heart disease plus stroke) over 10 years based on five risk factors: age, gender, smoking habit, systolic blood pressure, and the ratio of total cholesterol to HDL-cholesterol, modified from the original Framingham equations, to guide intervention and treatment targets [4]. However, no similar risk-stratification-risk-management programme for patients with HT had been established among Chinese.

The Risk Assessment and Management Programme–Hypertension (RAMP-HT) is an evidence-based [2, 4, 10], structured, protocol-driven multidisciplinary management programme launched by the HA in October 2011 to improve the quality of care (QOC) for HT patients in the public primary care setting. Hypertensive patients receiving care from GOPC, especially those who have suboptimal blood pressure control above 140/90 mmHg, are recruited. Standardized cardiovascular risk factor assessment, hypertensive complication screening and assessment on adherence to treatment are carried out on enrolled patients (Additional file 1). Patients are stratified into low, medium or high risk groups according to the 10-year cardiovascular disease (CVD) risk calculated from their relevant risk factors by the JBS 2005 Equation. A multi-disciplinary team comprised of doctors, nurses, dietitians, physiotherapist and/or occupational therapists then deliver individualized management targeted to the patient’s risk factors according to standardized risk-stratified guidelines (Additional file 2).

In order to facilitate the operation of RAMP-HT as an integrated component to usual GOPC care but not a stand-alone programme, multi-level organizational changes had been made. Patient assessment and education were delegated from frontline doctors to trained nurses and allied health professionals. A risk-guided management protocol was used to guide doctors and nurses regarding drug choices and referrals. A new clinical data entry platform was built to enhance relay of information between frontline doctors and other healthcare providers. These various strategies had all previously been proven to improve HT care [11], but few programmes incorporated as much changes and to such a scale [12–15].

The evaluation of QOC and effectiveness is an essential part of any chronic disease management programme in order to assure that the intended care is provided and whether a health benefit can be gained. The information will inform future policy and service planning. Therefore, we aim to evaluate the QOC and effectiveness of the RAMP-HT to prove if such approach of HT management can be implemented and beneficial in the naturalistic busy primary health care setting.

### Aims and objectives

The aim of this study is to evaluate the quality of care (QOC) and effectiveness of the multi-disciplinary Risk Assessment and Management Programme for Hypertension (RAMP-HT) for patients with HT managed by government-funded primary care clinics in Hong Kong. The evaluation is conducted using a structured and comprehensive evidence-based evaluation framework.

The objectives of the study are:

1. To review and identify the structure, process and outcome indicators of quality of care;
2. To identify the criterion and set the target standard for each indicator;
3. To compare the observed standards against the target standards;
4. To identify any on-site problems related to implementation of the RAMP-HT;
5. To provide feedback on the quality of care of the RAMP-HT;
6. To identify possible areas for improvement;
7. To give recommendations for enhancement of service delivery

The following hypotheses will be tested:

1. The structure and process criteria of care should be achieved up to standards by all participating clinics;
2. A higher proportion of patients should have achieved the target blood pressure and low density lipoprotein cholesterol (LDL-C) level after the RAMP-HT;
3. There should be a reduction in the predicted 10-year CVD risk 12 and 24 months after RAMP-HT;
4. Patients who have participated in the RAMP-HT should have better clinical outcomes than patients managed by usual-care (control);
5. RAMP-HT is associated with a lower incidence of cardiovascular diseases in the long term.

## Methods/Design

### Evaluation of quality of care

A longitudinal study using the Action Learning [16] and Audit Spiral methodologies [17] is conducted for a systematic analysis of the QOC and to identify areas for enhancement for the RAMP-HT. Two evaluation cycles are carried out with feedback of results and a quality enhancement action plan has been implemented after the first evaluation, aiming at a higher level of quality of care at the second cycle. Interim site visits are conducted to identify any problems of on-site implantation of the programme.

### Development of the evaluation framework

**Fig. 1:**
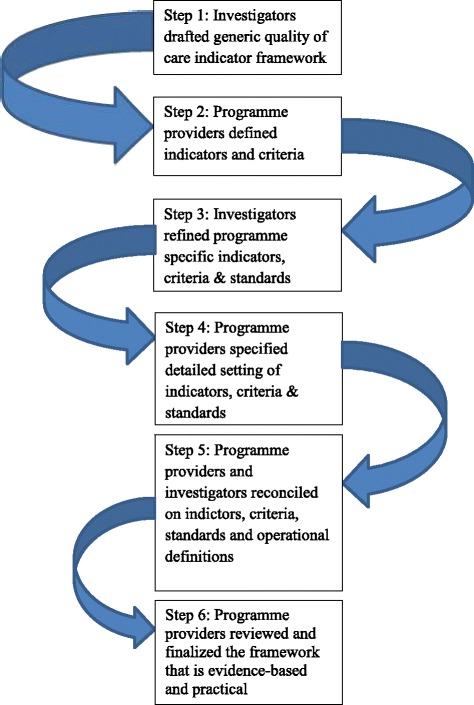
An iterative process and reconciliation between the investigators and the programme providers

Donabedian’s taxonomy of QOC on structure, processes and outcomes is used as the base of the evaluation framework [18]. A QOC framework has been developed by an iterative process and reconciliation between the investigators and the programme providers (Fig. 1). An intensive literature review on HT is first conducted to identify key elements of qualitied HT management in primary care. The investigators then work together with the programme providers (HA RAMP-HT Workgroup), frontline practitioners (HA doctors and nurses representatives) and the Statistics and Workforce Planning Department of the HA (HA statistics team) to discuss on which aspects need to be evaluated and what data are retrievable from the computer system. This final evaluation framework lists out the indicators of the structure (staff, facilities, organization and management), process (what, when and how care is delivered) and outcomes (clinical outcomes and patient reported outcomes) with the required criteria and standard of care to be achieved (Additional file 3).

### Subjects

All hypertensive patients without diabetes mellitus who are managed under the HA GOPC are referred from doctors and nurses to participate in the programme. Before enrolment to RAMP-HT, eligibility screening is performed to identify eligible patients based on the following inclusion and exclusion criteria: Inclusion criteria are patients who are diagnosed with hypertension and regularly followed up in GOPC; Patients are excluded if they are diagnosed with diabetes mellitus; they will be enrolled into another risk-stratification and management programme for diabetic patients. The priority of enrolment is reserved for patients who have suboptimal blood pressure control above 140/90 mmHg.

All patients enrolled into the RAMP-HT are included in the evaluation on process and clinical outcome of care for each evaluation cycle. To determine the clinical effectiveness of RAMP-HT, a matched control study is performed to compare RAMP-HT participants to matched HT patients receiving usual GOPC care who have not participated in the RAMP-HT. The same number of RAMP-HT participants and age-sex-disease severity-matched usual care HT patients are randomly selected. Disease severity is defined by the presence or absence of end-organ damages, disease control (baseline blood pressure level) and required treatment. It is not feasible to control for all co-morbidities but these will be examined as possible confounding factors in the data analysis.

A total of 600 patients, 300 RAMP-HT participants and 300 usual care HT patients, are recruited from GOPC by trained research assistants for a telephone survey on patient reported outcomes (PRO). All subjects who have given consent to the telephone follow-up survey are interviewed by telephone within 4 weeks and at 12 months from recruitment.

### Sample size calculation

To determine a difference between groups (RAMP-HT participants and usual-care group), proportions of 5 % in achieving the target clinical outcomes (blood pressure < 140/90 mmHg) with 80 % power and 95 % confidence interval, 1248 subjects are needed in each group [19]. Thus, a minimum of 1248 RAMP participants and 1248 controls has to be included in the evaluation of outcomes after 1 year.

The sample size for the evaluation of PRO score change after RAMP-HT is estimated to detect a minimally clinically important difference (MCID) that is equivalent to Cohen’s small effect size of 0.3 [20]. A sample size of 176 subjects in each group is required to have 80 % power and 95 % confidence interval to detect the expected difference by independent *t*-test [21]. Therefore, 300 patients in each group, i.e. a total of 600, have to be recruited to account for a maximum of 40 % dropouts.

### Data collection

#### Evaluation on structure and process

The coordinator of each participating HA cluster and clinic are asked to complete a structure of care questionnaire (Additional file 4). Provider characteristics are also collected. In addition to the self-assessment, we ask the provider to submit a list of the staff and facilities designated for the programme, and a description of the programme objectives and protocol. We also carry out site visits to cross-validate the data.

Anonymized data are retrieved from the computerized medical record system (CMS) by the HA statistics team to determine the patient recruitment rate, enrolment rate, risk stratification, attendance at RAMP-HT clinic, compliance with assessment and care per programme protocol, investigations and referral rates.

#### Evaluation on outcomes of care

Anonymized data of on the relevant clinical outcomes (e.g. blood pressure, LDL-C) of RAMP-HT subjects and selected usual-care comparison group at baseline, 12 and 24 months from enrolment are retrieved from the CMS by the HA statistics team. The 10-year predicted CVD risk of the RAMP-HT subjects and usual care groups recruited in both cycles is estimated base on the clinical parameters at 12 and 24 months from enrolment to evaluate the effectiveness of the programme in the reduction of predicted CVD risk.

RAMP-HT participants and usual care HT patients who have agreed to the PRO survey are interviewed by telephone by trained interviewers of the HKU Social Science Research Centre within 1 and at 12 months from enrolment to answer the Short Form-12 version 2 (SF-12v2) Health Survey on health-related quality of life (HRQOL), the Patient Enablement Instrument (PEI) and Global Rating of Change Scale (GRS) to detect change in health and a structured questionnaire on life-style measures and knowledge on hypertension (Additional file 5).

#### Two audit and feedback cycles

Two evaluation (audit) cycles on the standards of care are carried out at 24 months (September, 2013) and 48 months (September, 2015) from the start of the RAMP-HT programme. The first evaluation cycle includes subjects who are enrolled between October 2011 and March 2012 to evaluate the process of care and 12-month clinical outcomes at the start of the programme. The results have been fed back to the HA programme team to identify quality gaps and possible quality enhancement strategies. A revised QOC evaluation framework is developed for the second evaluation cycle based on new agreed indicators and target standards. The second evaluation cycle includes subjects enrolled into the RAMP-HT from January to June, 2014 to determine the standards that are achievable after the programme has been established. The results of second evaluation cycle will also be fed back to the programme team to identify any future areas for improvement of RAMP-HT.

### Outcome measures

Primary

1. The proportion of clinics that have satisfied each of the structure of care criteria.
2. The number and proportion of HT patients who have completed RAMP-HT.
3. The proportion of patients who have complied with the process of care criteria.
4. The proportion of patients who have achieved BP < 140/90 mmHg.
5. HT-related complication rates, e.g. coronary heart disease, cerebrovascular diseases, all-cause mortality, etc.

Secondary

1. Mean change in blood pressure, LDL-C level at 12 months
2. The predicted 10-year cardiovascular disease risk level stratified by RAMP-HT and 2008 Framingham prediction function at 12 and 24 months
3. Patient reported outcomes (PRO) measured by the change in SF-12v2 scores, the PEI and GRS scores at 12 months

### Study instruments

Study instruments are used in evaluating the structure of care and the PRO. Evaluation of the process of care and outcomes do not involve use of study instruments as the necessary data are retrieved from the HA through the Statistics and Workforce Planning Department.

### Structure of care questionnaire

Structure of care questionnaire is sent to the cluster co-ordinators and clinic doctors-in-charge of the RAMP-HT and requires their input. The questionnaire covers questions on resources spent on RAMP-HT, e.g. whether there is enough physical space and staff training for the programme, whether there is a data sharing platform within the programme, etc.

Three study instruments are used in the evaluation of the PRO, namely the short form-12 version 2 Health Survey, the Patient Enablement Instrument, and the Global Rating of Change Scale.

A. The Short Form-12 version 2 (SF-12v2) Health Survey. The Chinese (Hong Kong) SF-12v2 Health Survey is used to measure HRQOL. It has been validated [22] and normed [23] on the general Chinese population in Hong Kong. It measures eight domains of HRQOL on physical functioning, role physical, bodily pain, general health, vitality, social functioning, role emotional and mental health on a scale range from 0 to 100. A higher score indicates better HRQOL. The eight domain scores are aggregated into two summary scores, the physical (PCS) and mental (MCS) component summary.
B. The Patient Enablement Instrument (PEI). The PEI is a measure of patient’s enablement in coping with the illness and life [24]. It has 6 items each rated on a 3-point (0, 1, and 2) scale. The summation of the item scores gives the PEI score with a higher score indicating better enablement. The PEI has been translated into Chinese and is shown to be valid and reliable in Chinese patients in primary care [25].
C. The Global Rating of Change Scale (GRS). The GRS is adapted from those used in studies by Jaeschke and Osoba et al. [26]. It assesses the subject’s global perception of any change in the overall health condition on a 7-point scale (−3, −2, −1, 0, 1, 2, and 3) over the last 6 months.

### Data analysis

Descriptive statistics on standards of care are calculated by the percentage of clinics meeting the structure criteria, percentage of subjects enrolled, attendance and completion of the programme, receiving criterion process of intervention, investigations and referral per protocol and percentage of subjects achieving the criterion outcomes in each audit cycle. Cardiovascular risk analysis is performed by the Joint British Societies’ and the 2008 Framingham cardiovascular disease risk prediction functions. These equations predict the 10-year total risk of fatal and non-fatal CHD/CVD events and are applicable to patients aged 30–74 years. Within subject improvement in outcomes from baseline at 12 and at 24 months is analysed by paired *t*-test for continuous outcomes and McNemar test for binary outcomes. Differences in outcomes between RAMP-HT participants and usual-care group, between audit cycles are tested by analysis of variance (ANOVA) for continuous outcomes and Chi-squared tests for categorical outcomes. Self-selection biases are examined over inter-subject and inter-group differences by analysis of covariance. Multiple regressions are used to identify factors that are associated with quality of care or effectiveness.

### Ethics approvals

This study has received ethics approval from the Institutional Review Board of the University of Hong Kong/ Hospital Authority Hong Kong West (UW 13-227), Hong Kong East (HKEC-2013-029), Kowloon East and Kowloon Central (KC/KE-13-0069/ER-3), Kowloon West (KW/EX-13-082 (64-10)), New Territories East (CRE-2013.423), and New Territories West clusters (NTWC/CREC/1193/13).

### Funding

This study is part of the “Extended evaluation of quality of care of chronic disease management programmes and public-private partnership programmes of the Hospital Authority”, which was peer-reviewed and funded by the Health and Health Services Research Fund, Food and Health Bureau, HKSAR Commissioned Research on Enhanced Primary Care Study (Ref. no: EPC-HKU-2).

## Discussion

The multi-disciplinary RAMP-HT is a territory-wide quality improvement (QI) programme initiated by the Hospital Authority of Hong Kong to enhance HT care in the public primary care setting, which manages over 50 % of all HT patients in Hong Kong (according to HA internal data). This evaluation study differs from previous studies in that it is carried out in the naturalistic busy public primary care setting and is much larger in scale, which makes the results more valid and reliable. A participatory-action-research design is now recognized to be the most effective way to evaluate the quality of care of such a complex systematic intervention in the naturalistic clinical setting [27]. We engage all key stakeholders including frontline healthcare providers and the HA programme administrators right from the beginning to agree on the quality criteria and target standards and review the evaluation results in order to identify possible areas for quality enhancement and standards of good practice. The various parties are motivated to make changes in response to new evaluation evidence that emerges during the study process.

A similar evaluation study on Risk Assessment and Management Programme (RAMP) for diabetic patients was carried out in Hong Kong [28]. Fung et al. highlighted different practical aspects for conducting this type of action-research, namely coordination of stakeholder collaboration and communication, regular feedback and planning meetings, stakeholder endorsement and validation for the evaluation framework and operation definitions and ensuring data quality, which are also applicable to our RAMP-HT QOC evaluation.

The first evaluation cycle of our study has been completed in 2014. From our experience, the major challenges in this evaluation of quality of care study are:

1. Operational definitions of indicators of the evaluation framework
  Although indicators and criteria of qualitied HT care are well-documented in the literature and recommended in international and local management guidelines, we discovered variations in operational definitions among service providers. We have now agreed among all stakeholders on the explicit definitions of “blood pressure control” (i.e. averaged blood pressure readings over 6 months) and “target organ damage” (i.e. only left ventricular hypertrophy and stage 3 or above chronic kidney disease were included based on available data sources). The assessment methods for different target organ damages and cardiovascular complications (e.g. peripheral vascular disease) were found to vary across participating clinics, standardization on how to determine the presence of these conditions was essential. Some required clinical indicators were not captured systematically by existing data collection platform (e.g. presence of proteinuria by urine dipstick, ischaemic changes in the electrocardiogram) and thus could not be accurately evaluated, and our research team has to work with the frontline healthcare providers and programme administrators to decide on the best practices that are applicable and acceptable in the local setting.
  Setting appropriate target standard for each of the criterion in the evaluation framework is also important; there should be a balance between the ideal (normative) and what is practical (empirical) in the real life clinical setting. The standard needs to be set high enough to motivate quality improvement but low enough to be achievable.
2. Ensuring protocol adherence and data quality
  For evaluation of the quality of care, anonymized data on the processes and clinical outcomes of care are extracted from the Hospital Authority’s information system retrospectively. In order to cross-validate the accuracy of the data, it is essential to monitor the steps of which these data are generated. Site visits to different participating clinics to observe real-life practices have proven to be most informative. For instance, our team noticed that different definitions (pre-treatment versus post-treatment blood pressure or cholesterol levels) were used by frontline staff for CVD risk calculation; this issue was fed back to the RAMP-HT workgroup, leading to a consensus on using the pre-treatment levels. On the other hand, other variations such as staff or mode of patient education were considered acceptable because the same RAMP-HT operation principles were maintained.

The key to successful implementation of our evaluation study is the mutual trust among different partners being built up through open feedback and discussions. During an evaluation exercise, conflict of interest is inevitable: health policy decision makers concern about allocation of resources, programme administrators focus on service outcome while frontline healthcare providers may perceive it as an additional burden to their busy work. It is important to stress frequently that the ultimate collective aim of the study is quality improvement but not fault-finding. In addition, open channel communication is essential to allow adequate, effective discussions of concerns and practical difficulties among various parties. A good representation of key stakeholders consisting of frontline healthcare providers, programme planner and researchers must be included in the study workgroup who meet regularly throughout the study process from planning, implementing quality improvement strategies and dissemination of results.

### Conclusion

With the increasing numbers of patients with hypertension, a well-organized and comprehensive multi-disciplinary approach to care is the future direction of healthcare delivery. Proper evaluation is required to assure whether RAMP-HT can serve this purpose in real practice, and to provide evidence on its likely health benefit for the patients.

## Additional files

Additional file 1:
**Multi-disciplinary Risk Assessment & Management Programme–Hypertension (RAMP HT) workflow.**
Additional file 2:
**The standardized risk management guideline of RAMP-HT.**
Additional file 3:
**RAMP-HT evaluation of quality of care framework.**
Additional file 4:
**RAMP-HT structure of care questionnaire.**
Additional file 5:
**RAMP-HT patient reported outcomes questionnaire.**

## Abbreviations

RAMP-HT: Multi-disciplinary Risk Factor Assessment and Management Programme Hypertension
HT: Hypertension
QOC: Quality of Care
GOPCs: General Outpatient Clinics
BP: Blood Pressure
LDL-C: Low Density Lipoprotein-Cholesterol
CHD: Coronary Heart Disease
SF-12v2: SF-12 Health Survey Version 2
PEI: Patient Enablement Instrument
GRS: Global Rating of Change Scale
MCID: Minimally Clinically Important Difference
HRQOL: Health-related Quality of Life
PRO: Patient Reported Outcomes
CVD: Cardiovascular Disease
ANOVA: Analysis of Variance
