# Supplementary material for: Evaluation of the quality of care of a multi-disciplinary Risk Factor Assessment and Management Programme for Hypertension (RAMP-HT)
Source: BMC Fam Pract. 2015 Jun 19;16:71. doi: 10.1186/s12875-015-0291-0 (PMC4471929; doi:10.1186/s12875-015-0291-0)
Supplement: Additional file 1: — Multi-disciplinary Risk Assessment & Management Programme–Hypertension (RAMP HT) workflow. [file 12875_2015_291_MOESM1_ESM.docx]

Additional file 1: Multi-disciplinary Risk Assessment & Management Programme – Hypertension (RAMP HT) Workflow

| **Recruitment**  **Criteria** | - Hypertensive patients with regular follow up in GOPC   (Priority group: patients with blood pressure ≥ 140/90 mmHg)  AND   - Without DM (Patients with DM should be referred to RAMP-DM) | **Doctors** |
| --- | --- | --- |

| **Comprehensive Assessment** | | |
| --- | --- | --- |
| **Basic**  **Parameters** | - Blood pressure and pulse - Body weight, body height, and BMI - Waist circumference | **PCA** |
| **Laboratory Tests** | - Blood tests for Fasting Glucose, Lipid Profile and Renal Function Tests - Urine for protein (ACR optional) - Electrocardiogram (if not done before) | **PCA** |

| **Risk Stratification** | **10 Years’ Cardiovascular Disease Risk Calculation Based on JBS 2005 Equation**  Patient data for CVD risk calculation:  (1) Age (2) Sex (3) Total Cholesterol (4) Smoking status  (5) Pre-treatment systolic BP (Use 160mmHg if not known)  **Exception: Patients with existing CVD will be classified as High Risk* | **Nurse** |
| --- | --- | --- |

| **Risk Explanation and Education** | **Risk Explanation and Education**   - Explanation of risk level - Coordination of risk management - Lifestyle Modification | **Nurse +/- PEP** |
| --- | --- | --- |

| **Low Risk (<10%)** | **Medium Risk (10-20%)** | **High Risk (>20%)** |
| --- | --- | --- |

| Consider RAMP-HT Clinic and nurse and allied health clinics referral according to  cluster-based guidelines |
| --- |
