# Supplementary material for: Evaluation of the quality of care of a multi-disciplinary Risk Factor Assessment and Management Programme for Hypertension (RAMP-HT)
Source: BMC Fam Pract. 2015 Jun 19;16:71. doi: 10.1186/s12875-015-0291-0 (PMC4471929; doi:10.1186/s12875-015-0291-0)
Supplement: Additional file 2: — The standardized risk management guideline of RAMP-HT. [file 12875_2015_291_MOESM2_ESM.docx]

Additional file 2: The standardized risk management guideline of RAMP-HT

The RAMP-HT risk management pathway

| **Blood pressure** | **Low Risk**  **(<10%)** | **Medium Risk**  **(10-20%)** | **High Risk**  **(>20%)** |
| --- | --- | --- | --- |
| 140/90 – 159/99mmHg | GOPC | GOPC | GOPC  + statin if  LDL-C suboptimal |
| ≥ 160/100 mmHg | GOPC Dr ± RAMP-HT Nurse | | |
| ≥ 160/100 mmHg  +  On ≥ 3 kinds of anti HT drugs | RAMP-HT Nurse  +  Family medicine specialist | | |
| **Other interventions:**  **Patient Empowerment Programme** – offer to all HT patients  **Smoking Counselling and Cessation Programme/Centre** – Smoker intend to quit  **Dietitian** – (1) BMI ≥ 27.5kg/m^2^ for weight reduction or, (2) special dietary needs or, (3) poor dietary control despites nursing intervention offered  **Physiotherapist** – BMI ≥ 27.5kg/m^2^ intend to join weight reduction programme  **Integrated Mental Health Programme and/or Occupational therapist** – presence of emotional problems | | | |

GOPC: General Out-patient clinic
