# Supplementary material for: Evaluation of the quality of care of a multi-disciplinary Risk Factor Assessment and Management Programme for Hypertension (RAMP-HT)
Source: BMC Fam Pract. 2015 Jun 19;16:71. doi: 10.1186/s12875-015-0291-0 (PMC4471929; doi:10.1186/s12875-015-0291-0)
Supplement: Additional file 3: — RAMP-HT evaluation of quality of care framework. [file 12875_2015_291_MOESM3_ESM.docx]

Additional file 3: RAMP-HT Evaluation of Quality of Care Framework

Risk Assessment & Management Programme

– Hypertension (RAMP-HT)

**Evaluation of Quality of Care Framework**

**Structure of Care (1)**

| **Human Resources** | | **Target  Standard** |
| --- | --- | --- |
| 1. There **must** be designated programme coordinator(s) to oversee the RAMP-HT at cluster level. | | 100% |
|  | | |
| 1. There **must** be a multidisciplinary team of health care personnel, including FM specialist(s), advanced practice nurse(s)/ registered nurse(s), allied health professional(s) and health care supporting staff to implement the RAMP-HT. | | 100% |
|  | | |
| 1. Allied health professional(s) (e.g. dieticians, physiotherapists etc.) **should** be accessible to patients in the RAMP-HT when indicated. | | 50% |
|  | | |
| 1. RAMP-HT team staff **must** be familiar with the programme objectives and logistics. | | 100% |
|  | | |
| 1. RAMP-HT team doctors and nurses **must** know the management protocol. | | 100% |
|  | | |
| 1. RAMP-HT team staff **should** have undergone relevant training for this programme. | 70% |  |
|  | |  |

**Structure of Care (2)**

| **Office Infrastructure** | **Target**  **Standard** |
| --- | --- |
| 1. FM Module in CMS **must** be used for documentation of patient data. | 100% |
|  | |
| 1. Professional staff of the RAMP-HT team **must** have access to the CMS system for patient data entry, sharing and retrieval. | 100% |
|  | |
| 1. There **should** be appropriate physical space provided for the programme. | 70% |
|  | |
| 1. Equipment and laboratory service for the programme **should** be available in the clinic.   List of essential equipment and laboratory service:   1. Blood pressure measurement device 2. ECG machine 3. Urine dipstick for protein or access to laboratory service with urine protein analysis capability 4. Access to laboratory service for: a) renal function test, b) lipid profile, c) fasting glucose or oral glucose tolerance test | 70% |
|  | |
| 1. Educational materials on disease knowledge **must** be available. | 100% |
|  | |

**Structure of Care (3)**

| **Programme Management & Organizational Structure** | **Target**  **Standard** |
| --- | --- |
| 1. Patients enrolled in the RAMP-HT **must** be properly documented in the CMS / OPAS. | 100% |
|  | |
| 1. Patient enrolment records (e.g. in CMS / OPAS) **must** be accessible to the doctors and other authorized members of the RAMP-HT team. | 100% |
|  | |
| 1. The patient's doctor **should** be informed of or have access to patient’s participation in RAMP-HT and cardiovascular risk stratification. | 80% |
|  | |
| 1. There **should** be regular meetings among staff of each participating RAMP-HT team to monitor the performance of the programme. | 70% |
|  | |
| 1. There **should** be regular meetings between RAMP-HT team staff and the cluster programme coordinator(s). | 70% |
|  | |

**Process of Care (1)**

| **Service Delivery & Process of Care (1)** | | **Target  Standard** |
| --- | --- | --- |
| 1. HT patients without DM **could** be enrolled^a^ into the RAMP-HT. | | N=4200  Patients/  team/year |
|  | | |
| 1. Patients with BP ≥140/90mmHg AND aged < 80 year **should** be enrolled into RAMP-HT. | | 60% |
|  | | |
| 1. All enrolled patients **must** complete the RAMP-HT intake assessment within 1 year before the first RAMP attendance.   List of essential assessment/procedures: | |  |
| 1. Blood pressure | | 100% |
| 1. BMI | | 100% |
| 1. Waist circumference | | 100% |
| 1. Smoking status | | 100% |
| 1. CVD risk | | 100% |
| 1. Drug compliance | | 80% |
| 1. Peripheral vascular disease | | 80% |
| 1. Exercise | | 80% |
| 1. Diet | | 80% |
|  | |  |

^a^ Ever attended to RAMP-HT

**Process of Care (2)**

| **Service Delivery & Process of Care (2)** | **Target  Standard** |
| --- | --- |
| 1. All enrolled patients **should** have blood test for lipid profile in the past 1 year. | 80% |
|  | |
| 1. All enrolled patients **should** have blood test for fasting glucose or oral glucose tolerance test (OGTT) in the past 1 year. | 80% |
|  | |
| 1. All enrolled patients **should** have blood test for creatinine in the past 1 year. | 80% |
|  | |
| 1. All enrolled patients **should** have urine test for protein in the past 1 year. | 80% |
|  | |

**Process of Care (3)**

| **Service Delivery & Process of Care (3)** | | **Target  Standard** |
| --- | --- | --- |
| 1. All enrolled patients **should** have an ECG at enrolment or ever done with results on record. | | 50% |
|  | | |
| 1. All enrolled patients without target organ damage or existing CVD within one year before the first RAMP attendance **must** be stratified into a cardiovascular risk group based on the JBS 2005 Equation. | | 95% |
|  | | |
| 1. All enrolled patients with target organ damage (TOD) or existing CVD within one year before the first RAMP attendance **should** be classified as high risk. | | 80% |
|  | |  |

**Process of Care (4)**

| **Service Delivery & Process of Care (4)** | | **Target  Standard** |
| --- | --- | --- |
| 1. Patients who smoke **could** be referred to smoking counselling and cessation centres (SCCC). | | 20% |
|  | | |
| 1. The use of home-blood pressure monitoring **must** be assessed for all patients. | | 100% |
|  | | |
| 1. High risk patients with *suboptimal risk factors (based on the latest BMI, LDL and BP results within one year before the first RAMP attendance) **should** attend at least one nurse clinic session.   *Definition of suboptimal risk factors: BMI ≥ 25 AND;  LDL ≥ 2.6 mmol/L AND;  BP ≥ 140/90 mmHg | | 70% |
|  | | |
| 1. Patients with SBP≥160mmHg AND DBP≥100mmHg AND on ≥3 kinds of anti-hypertensive drugs **should** attend at least one RAMP-HT doctor consultation. | | 70% |
|  | |  |

**Outcomes of Care (1)**

| **Clinical Outcomes** | **Target  Standard** |
| --- | --- |
| 1. Patients **should** have clinic BP < 140/90 mmHg at least one year after the programme. | 65% |
|  | |
| 1. Patients **could** have improvement in BP one year after the programme. | Improvement in mean Systolic BP (mmHg) |
|  | Improvement in mean Diastolic BP (mmHg) |
|  | |
| 1. Patients with high CVD risk **should** have LDL-C < 2.6mmol/L one year after the programme. | 70% |
|  | |
| 1. Patients with high CVD risk not at target (LDL-C ≥ 2.6 mmol/L) **could** have improvement in LDL-C one year after the programme. | Improvement in mean  LDL-C (mmol/L) |
|  | |

**Outcomes of Care (2)**

| **Patient Reported Outcomes** | **Target  Standard**  **(% of patients)** |
| --- | --- |
| 1. Patients **should** have no deterioration in quality of life (SF-12) after the programme. | No deterioration |
|  | |
| 1. Patients **should** have stable or improved health measured by Global Rating Scale after the programme. | 60% |
|  | |
| 1. Patients **should** be more enabled (Patient Enablement Instrument) after the programme. | 60% |
|  | |
| 1. Patients **should** have improvement in knowledge on hypertension after the programme. | 70% |
|  | |
| 1. Patients **should** perform self-blood pressure monitoring. | 50% |
|  | |
