# Supplementary material for: Evaluation of the quality of care of a multi-disciplinary Risk Factor Assessment and Management Programme for Hypertension (RAMP-HT)
Source: BMC Fam Pract. 2015 Jun 19;16:71. doi: 10.1186/s12875-015-0291-0 (PMC4471929; doi:10.1186/s12875-015-0291-0)
Supplement: Additional file 4: — RAMP-HT structure of care questionnaire. [file 12875_2015_291_MOESM4_ESM.docx]

**Additional file 4: RAMP-HT Structure of care questionnaire**

**Risk Assessment & Management Programme –**

**Hypertension (RAMP-HT)
Structure of Care Questionnaire**

**for Cluster Level**

| **Note:**   1. This questionnaire should be **completed by the cluster coordinator of the programme** 2. For supporting material included as attachments, please kindly **specify the corresponding question number on the top right hand corner of each page** |
| --- |

| **Cluster** |  |  |  |  |
| --- | --- | --- | --- | --- |

| **Completed Date (DD/MM/YYYY)** |  |  | / |  |  | / |  |  |  |  |
| --- | --- | --- | --- | --- | --- | --- | --- | --- | --- | --- |

Please complete each question by **circling your response or deleting the inappropriate answers**. Please also **fill the appropriate information in the blank.**

| **Section A: Human Resources** | | | | | |
| --- | --- | --- | --- | --- | --- |
| 1 | Is there a designated programme coordinator to oversee the RAMP-HT at cluster level in your cluster? | 1. Yes | 2. No | 3. Not sure | |
| 2 | Is there a multidisciplinary team of healthcare personnel, including Family Medicine (FM) Specialist(s), Advanced Practice Nurse (s)/ Registered Nurse (s), Allied Health Professional(s) and health care supporting staff to implement RAMP-HT in your cluster? | 1. Yes | 2. No | 3. Not sure | |
| Please indicate the total number of the following healthcare personnel and the percentage of their time dedicated to RAMP-HT in your cluster: *(Put down ZERO if not applicable)*  _____ full-time FM Specialist(s); each allocate _____% of their time.  _____ part-time FM Specialist(s); each allocate _____% of their time.  _____ full-time Advanced Practice Nurse(s); each allocate _____% of their time. _____ part-time Advanced Practice Nurse(s); each allocate _____% of their time.  _____ full-time Registered Nurse(s); each allocate _____% of their time. _____ part-time Registered Nurse(s); each allocate _____% of their time.  _____ full-time Allied Health Professional(s); each allocate _____% of their time.  _____ part-time Allied Health Professional(s); each allocate _____% of their time.  _____ full-time Health care supporting staff; each allocate _____% of their time.  _____ part-time Health care supporting staff; each allocate _____% of their time.  Others: | | | | | |
|  | | | | | |
| 3 | Are Allied Health Professionals (e.g. Dietitians, Physiotherapists etc.) accessible to patients in the RAMP-HT when indicated in your cluster? | 1. Yes | 2. No | 3. Not sure | |
| 4. | Are you and your RAMP-HT team staff familiar with the programme objectives and logistics in your cluster?  *(“familiar”: able to give an overview of the programme objectives and logistics.)*  ***Please attach a summary and mark “Q4” on the top right hand corner.****)* | 1. Yes | 2. No | 3. Not sure | |
| 5 | Do you and your RAMP-HT team doctors and nurses know the management protocol of the RAMP-HT in your cluster?  *(“know”: read and understood the programme management protocol.* ***Please attach a summary and mark “Q5” on the top right hand corner.****)* | 1. Yes | 2. No | 3. Not sure | |
| 6 | Have the RAMP-HT team staff undergone relevant training for this programme in your cluster? | 1. Yes | 2. No | 3. Not sure | |
| Please specify what training has been taken: | | | | | |
| **Section B: Office Infrastructure** | | | | | |
| 7 | Is FM Module in CMS used for documentation of patient data in your cluster?  (***Please attach a sample copy or print screen of all forms, and mark “Q7” on the top right hand corner.****)* | 1. Yes | 2. No | 3. Not sure | |
| 8 | Do the professional staff of your RAMP-HT team(s) have access to the CMS for patient data entry, sharing and retrieval in your cluster?  *(****Please enclose a sample copy of relevant print screens, and mark “Q8” the top right hand corner.****)* | 1. Yes | 2. No | 3. Not sure | |
| 9 | Is there appropriate physical space provided for the programme in your cluster? | 1. Yes | 2. No | 3. Not sure | |
| 10 | Do all the RAMP-HT clinics in your cluster have the following equipment and laboratory service available for the RAMP-HT programme? | | | | |
|  | 1. Blood pressure measurement device? | 1. Yes | 2. No | 3. Not sure | |
|  | 1. ECG machine? | 1. Yes | 2. No | 3. Not sure | |
|  | 1. Urine dipstick for protein or access to laboratory service with urine protein analysis capability | 1. Yes | 2. No | 3. Not sure | |
|  | 1. Access to laboratory service for: |  |  |  | |
|  | - 1. Renal function tests? | 1. Yes | 2. No | 3. Not sure | |
|  | - 1. Lipid profile? | 1. Yes | 2. No | 3. Not sure | |
|  | - 1. Fasting glucose or oral glucose tolerance test? | 1. Yes | 2. No | 3. Not sure | |
| 11 | Are educational materials on disease knowledge available in all the RAMP-HT clinics in your cluster?  *(****Please enclose a copy of relevant materials and mark “Q11” on the top right hand corner.****)* | 1. Yes | 2. No | 3. Not sure | |
| **Section C: Programme Management & Organizational Structure** | | | | | |
| 12 | Are patients enrolled in the RAMP-HT in your cluster properly documented in the CMS/OPAS?  *(****Please enclose a sample copy of relevant print screens and mark “Q12” on the top right hand corner.****)* | 1. Yes | 2. No | 3. Not sure | |
| 13 | Are patient enrolment records (e.g. in CMS/ OPAS) accessible to the doctors and other authorized members of the RAMP-HT team in your cluster? | 1. Yes | 2. No | 3. Not sure | |
| 14 | Is the patient’s doctor in your cluster being informed of or do they have access to their patients’ participation in RAMP-HT and cardiovascular risk stratification?  If yes, how are they informed or how do they access? | 1. Yes | 2. No | 3. Not sure | |
|  | 1. Reminder | 1. Yes | 2. No | 3. Not sure | |
|  | 1. Record in the CMS | 1. Yes | 2. No | 3. Not sure | |
|  | 1. Patients’ hand-held record | 1. Yes | 2. No | 3. Not sure | |
|  | 1. Others, please specify: | 1. Yes | 2. No | 3. Not sure | |
| 15 | Are there regular meetings among staff of each participating RAMP-HT team(s) to monitor the performance of the programme in your cluster? | 1. Yes | 2. No | 3. Not sure | |
| Please specify the frequency and form of communication related to the RAMP-HT: | | | | | |
| 16 | Are there regular meetings between RAMP-HT team staff, and the cluster programme coordinator(s) in your cluster? | 1. Yes | 2. No | 3. Not sure | |
| Please specify the frequency and form of communication related to the RAMP-HT: | | | | | |

**Risk Assessment & Management Programme –**

**Hypertension (RAMP-HT)
Structure of Care Questionnaire**

**for Clinic Level**

| **Note:**   1. This questionnaire should be **completed by the person-in-charge of the RAMP-HT programme in the clinic**. Each clinic should have an individual questionnaire. In order words, if the person takes responsibility for more than one clinic, he/she is required to fill in more than one questionnaire, for each clinic separately. 2. For supporting material included as attachments, please kindly **specify the corresponding question number on the top right hand corner of each page**. |
| --- |

| **Cluster** |  |  |  |  | **Clinic** |  |  |  |  |
| --- | --- | --- | --- | --- | --- | --- | --- | --- | --- |

| **Completed Date (DD/MM/YYYY)** |  |  | / |  |  | / |  |  |  |  |
| --- | --- | --- | --- | --- | --- | --- | --- | --- | --- | --- |

Please complete each question by **circling your response or deleting the inappropriate answers**. Please also **fill the appropriate information in the blank**.

| **Section A: Human Resources** | | | | |
| --- | --- | --- | --- | --- |
| 1 | Is there a designated programme coordinator(s) to oversee the RAMP-HT in your clinic? | 1. Yes | 2. No | 3. Not sure |
| 2 | Is there a multidisciplinary team of healthcare personnel, including Family Medicine (FM) Specialist(s), Advanced Practice Nurse(s)/ Registered Nurse(s), Allied Health Professional(s) and health care supporting staff to implement the RAMP-HT in your clinic? | 1. Yes | 2. No | 3. Not sure |
| Please indicate the number of the following healthcare personnel and the percentage of their time dedicated to RAMP-HT in your clinic: *(Put down ZERO if not applicable)*  _____ full-time FM Specialist(s); each allocate _____% of their time.  _____ part-time FM Specialist(s); each allocate _____% of their time.  _____ full-time Advanced Practice Nurse(s); each allocate _____% of their time. _____ part-time Advanced Practice Nurse(s); each allocate _____% of their time.  _____ full-time Registered Nurse(s); each allocate _____% of their time. _____ part-time Registered Nurse(s); each allocate _____% of their time  _____ full-time Allied Health Professional(s); each allocate _____% of their time.  _____ part-time Allied Health Professional(s); each allocate _____% of their time.  _____ full-time Health care supporting staff; each allocate _____% of their time.  _____ part-time Health care supporting staff; each allocate _____% of their time.  Others: | | | | |
| 3 | Are Allied Health Professionals (Dietitians and Physiotherapists etc.) accessible to patients in the RAMP-HT when indicated in your clinic? | 1. Yes | 2. No | 3. Not sure |
| 4 | Are you and your RAMP-HT team staff familiar with the programme objectives and logistics in your clinic?  *(“familiar”: able to give an overview of the programme objectives and logistics.)* ***Please attach a summary and mark “Q4” on the top right hand corner****)* | 1. Yes | 2. No | 3. Not sure |
| 5 | Do you and your RAMP-HT team doctors and nurses know the management protocol of the RAMP-HT in your clinic?  *(“know”: read and understood the programme management protocol.* ***Please attach a summary and mark “Q5” on the top right hand corner****)* | 1. Yes | 2. No | 3. Not sure |
| 6 | Have the RAMP-HT team staff undergone relevant training for this programme in your clinic? | 1. Yes | 2. No | 3. Not sure |
| Please specify what training has been taken: | | | | |
| **Section B: Office Infrastructure** | | | | |
| 7 | Is FM Module in CMS used for documentation of patient data in your clinic?  *(****Please enclose a sample copy or print screen of all forms, and mark “Q7” on the top right hand corner****)* | 1. Yes | 2. No | 3. Not sure |
| 8 | Do the professional staff of your RAMP-HT team(s) have access to the CMS for patient data entry, sharing and retrieval in your clinic?  *(****Please enclose a sample copy of relevant print screens, and mark “Q8” on the top right hand corner****)* | 1. Yes | 2. No | 3. Not sure |
| 9 | Is there appropriate physical space provided for the programme in your clinic? | 1. Yes | 2. No | 3. Not sure |

| 10 | Does your clinic have the following equipment and laboratory service available for the RAMP-HT programme? | | | |
| --- | --- | --- | --- | --- |
|  | 1. Blood pressure measurement device? | 1. Yes | 2. No | 3. Not sure |
|  | 1. ECG machine? | 1. Yes | 2. No | 3. Not sure |
|  | 1. Urine dipstick for protein or access to laboratory service with urine protein analysis capability? | 1. Yes | 2. No | 3. Not sure |
|  | 1. Access to laboratory service for: |  |  |  |
|  | - 1. Renal function tests? | 1. Yes | 2. No | 3. Not sure |
|  | - 1. Lipid profile? | 1. Yes | 2. No | 3. Not sure |
|  | - 1. Fasting glucose or oral glucose tolerance test? | 1. Yes | 2. No | 3. Not sure |
| 11 | Are educational materials on disease knowledge available in your clinic?  *(****Please enclose a copy of relevant materials and mark “Q11” on the top right hand corner.****)* | 1. Yes | 2. No | 3. Not sure |
| **Section C: Programme Management & Organizational Structure** | | | | |
| 12 | Are patients enrolled in the RAMP-HT in your clinic properly documented in the CMS/OPAS?  *(****Please enclose a sample copy of relevant print screens and mark “Q12” on the top right hand corner.****)* | 1. Yes | 2. No | 3. Not sure |
| 13 | Are patient enrolment records (e.g. in CMS/ OPAS) accessible to the doctors and the other authorized members of the RAMP-HT team in your clinic? | 1. Yes | 2. No | 3. Not sure |
| 14 | Is the patient’s doctor in your clinic being informed of or do they have access to their patients’ participation in RAMP-HT and cardiovascular risk stratification?  If yes, how are they informed or how do they access? | 1. Yes | 2. No | 3. Not sure |
|  | a) Reminder | 1. Yes | 2. No | 3. Not sure |
|  | b) Record in the CMS | 1. Yes | 2. No | 3. Not sure |
|  | c) Patients’ hand-held record | 1. Yes | 2. No | 3. Not sure |
|  | 1. Others, please specify: | 1. Yes | 2. No | 3. Not sure |
| 15 | Are there regular meetings among staff of each participating RAMP-HT team(s) to monitor the performance of the RAMP in your clinic? | 1. Yes | 2. No | 3. Not sure |
| Please specify the frequency and form of communication related to the RAMP-HT: | | | | |
| 16 | Are there regular meetings between RAMP-HT team staff, and the cluster programme coordinator(s) in your clinic? | 1. Yes | 2. No | 3. Not sure |
| Please specify the frequency and form of communication related to the RAMP-HT: | | | | |
