# Supplementary material for: Evaluation of the quality of care of a multi-disciplinary Risk Factor Assessment and Management Programme for Hypertension (RAMP-HT)
Source: BMC Fam Pract. 2015 Jun 19;16:71. doi: 10.1186/s12875-015-0291-0 (PMC4471929; doi:10.1186/s12875-015-0291-0)
Supplement: Additional file 5: — RAMP-HT patient reported outcomes questionnaire. [file 12875_2015_291_MOESM5_ESM.docx]

**Additional file 5: RAMP-HT Patient reported outcomes questionnaire**

**Baseline Survey**

**SF-12 HEALTH SURVEY (VERSION 2.0)**

INSTRUCTIONS: This survey asks for your views about your health. This information will help keep track of how you feel and how well you are able to do your usual activities.

Answer every question by marking the answer as indicated. If you are unsure about how to answer a question, please give the best answer you can.

1. In general, would you say your health is:

| Excellent | Very good | Good | Fair | Poor |  |
| --- | --- | --- | --- | --- | --- |
| 1 | 2 | 3 | 4 | 5 | |

2. The following questions are about activities you might do during a typical day. Does **your health now limit you** in these activities? If so, how much?

|  | Yes, limited a lot | Yes, limited a little | No, not limited at all |
| --- | --- | --- | --- |
| a. **Moderate activities**, such as moving a table, pushing a vacuum cleaner, bowling, or playing golf | 1 | 2 | 3 |
| b. Climbing **several** flights of stairs | 1 | 2 | 3 |

3. During the **past 4 weeks**, how much of the time have you had any of the following problems with your work or other regular daily activities **as a result** **of your physical health**?

|  | All of the time | Most of the time | Some of the time | A little of the time | None of the time |
| --- | --- | --- | --- | --- | --- |
| a. **Accomplished less** than you would like | 1 | 2 | 3 | 4 | 5 |
| b. Were limited in the **kind** of work or other activities | 1 | 2 | 3 | 4 | 5 |

4. During the **past 4 weeks**, how much of the time have you had any of the following problems with your work or other regular daily activities **as a result of any emotional problems** (such as feeling depressed or anxious)?

|  | All of the time | Most of the time | Some of the time | A little of the time | None of the time |
| --- | --- | --- | --- | --- | --- |
| a.　**Accomplished less** than you would like | 1 | 2 | 3 | 4 | 5 |
| b.　Did work or other activities less **carefully** than usual | 1 | 2 | 3 | 4 | 5 |

5. During the **past 4 weeks**, how much did **pain** interfere with your normal work (including both work outside the home and housework)?

| Not at all | A little bit | Moderately | Quite a bit | Extremely |
| --- | --- | --- | --- | --- |
| 1 | 2 | 3 | 4 | 5 |

6. These questions are about how you feel and how things have been with you during the **past 4 weeks**. For each question, please give the one answer that comes closest to the way you have been feeling. How much of the time during the **past 4 weeks**...

|  | All of the time | Most of the time | Some of the time | A little of the time | None of the time |
| --- | --- | --- | --- | --- | --- |
| a. have you felt calm and peaceful? | 1 | 2 | 3 | 4 | 5 |
| b. did you have a lot of energy? | 1 | 2 | 3 | 4 | 5 |
| c. have you felt downhearted and depressed? | 1 | 2 | 3 | 4 | 5 |

7. During the **past 4 weeks**, how much of the time has your **physical health** or **emotional problems** interfered with your social activities (like visiting friends, relatives, etc.)?

| All of the time | Most of the time | Some of the time | A little of the time | None of the time |  |
| --- | --- | --- | --- | --- | --- |
| 1 | 2 | 3 | 4 | 5 | |

**Part I. Knowledge on Hypertension:**

1. **Target blood pressure control should be:**

Higher than 140 / 90mmHg

Not higher than 140 / 90mmHg

Lower than 90 / 60mmHg

Don’t know

1. **If suspected side effects occur after taking anti-hypertensive drugs, what should I do?**

Stop taking the drugs immediately

Switch to previous drugs immediately

Continue to take drugs according to doctor’s prescription; if the side effects continue, then revisit the doctor as soon as possible

Reduce the dose of medication by myself

Don’t know

1. **Which of the following exercise should be avoided by hypertensive patients?**

Sprint

Tai chi

Walking

Stretching exercise

Don’t know

1. **Which of the followings is a healthy lifestyle for hypertensive patients?**

Pursue exciting activities

Smoke, drink, have strong tea and coffee

Have enough sleep and a regular schedule

Don’t know

1. **Which of the following food is rich in salt?**

Fresh pork

Luncheon meat

Steamed fish

Don’t know

**Part II. Lifestyle**:

1. **Do/did you smoke?**

Never

Ex-smoker: I used to smoke ____cigarettes every day, have quitted for ____ year(s) ____ month(s)

Current smoker: I smoke _____ cigarettes every day, for ____ year(s) ____ month(s)

1. **Do/did you drink?**

Never

Ex-drinker: I have stopped drinking completely for ____ year(s) ____ month(s)

Current drinker: Type of alcohol: _________; _______ units / week on average

Current drinker: in the past month, I have drunk in a single occasion more than 5 cans of beer / 5 glasses of dinner wine / 5 shots of liquor for ____ times

1. **Diet habit:**
   1. I take ______ fruits every day on average. (please input number of fruits taken)
   2. I take ______ portions of vegetables every day on average (1 portion equals half a bowl).
   3. I take food like salted meat, preserved meat, salted eggs or preserved bean curd ____ times in a week on average.
2. **Do you exercise regularly?**

No

Yes, _______ times every week, _______ minutes each time;

Type of exercise: _________________

1. **Self blood pressure monitoring:**
   1. How often do you monitor blood pressure?

Never (end of questionnaire)

Regularly, _______ times each month

Irregularly, _______ times each month

- 1. Where do you monitor your blood pressure usually?

At home

In community centres

Other sites, please specify: ________________

- 1. Would you adjust the following interventions by yourself according to your blood pressure readings?

No

Adjust the dose of medication

Adjust diet

Adjust exercise

**Part III: Demographic data:**

| 11. What is your marital status? | 1. Single | | | 2. Married | | | | | | 3. Separated or divorced | | | 4. Widowed | | | | | 5. Refuse to answer | |  |
| --- | --- | --- | --- | --- | --- | --- | --- | --- | --- | --- | --- | --- | --- | --- | --- | --- | --- | --- | --- | --- |
|  |  | | | |  | | |  | | | | | |  | | |  | | |  |
| 12. What is the average monthly income of your household from all sources?  $________________ | 1.  Less than $2,000 | 2.  $2,000  \|  $3,999 | | | | 3.  $4,000  \|  $5,999 | 4.  $6,000  \|  $7,999 | | | | 5.  $8,000  \|  $9,999 | | | | 6.  $10,000  \|  $14,999 | 7.  $15,000  \|  $19,999 | | | 8.  $20,000  \|  $24,999 | |
|  | 9.  $25,000  \|  $29,999 | 10.  $30,000  \|  $39,999 | | | | 11.  $40,000  \|  $59,999 | 12.  $60,000  or  above | | | | 13.  No income | | | | 14.  Refuse to  answer | 15.  Don’t know | | |  | |
|  |  | | | |  | | |  | | | | | |  | | |  | | | |
| 13. What is your average monthly income, including all sources?  $________________ | 1.  Less than $2,000 | | 2.  $2,001  \|  $5,000 | | | | | | 3.  $5,001  \|  $9,999 | | | 4.  $10,000  \|  $14,999 | | | | | | 5.  $15,000  \|  $19,999 | | |
|  | 6.  $20,000  \|  $29,999 | | 7.  $30,000  or  above | | | | | | 8.  No income | | | 9.  Refuse to answer | | | | | | 10.  Don’t know | | |

------------------------------------------------- End ---------------------------------------

**Follow-up Survey**

**SF-12 HEALTH SURVEY (VERSION 2.0)**

INSTRUCTIONS: This survey asks for your views about your health. This information will help keep track of how you feel and how well you are able to do your usual activities.

Answer every question by marking the answer as indicated. If you are unsure about how to answer a question, please give the best answer you can.

1. In general, would you say your health is:

| Excellent | Very good | Good | Fair | Poor |  |
| --- | --- | --- | --- | --- | --- |
| 1 | 2 | 3 | 4 | 5 | |

2. The following questions are about activities you might do during a typical day. Does **your health now limit you** in these activities? If so, how much?

|  | Yes, limited a lot | Yes, limited a little | No, not limited at all |
| --- | --- | --- | --- |
| a. **Moderate activities**, such as moving a table, pushing a vacuum cleaner, bowling, or playing golf | 1 | 2 | 3 |
| b. Climbing **several** flights of stairs | 1 | 2 | 3 |

3. During the **past 4 weeks**, how much of the time have you had any of the following problems with your work or other regular daily activities **as a result** **of your physical health**?

|  | All of the time | Most of the time | Some of the time | A little of the time | None of the time |
| --- | --- | --- | --- | --- | --- |
| a. **Accomplished less** than you would like | 1 | 2 | 3 | 4 | 5 |
| b. Were limited in the **kind** of work or other activities | 1 | 2 | 3 | 4 | 5 |

4. During the **past 4 weeks**, how much of the time have you had any of the following problems with your work or other regular daily activities **as a result of any emotional problems** (such as feeling depressed or anxious)?

|  | All of the time | Most of the time | Some of the time | A little of the time | None of the time |
| --- | --- | --- | --- | --- | --- |
| a.　**Accomplished less** than you would like | 1 | 2 | 3 | 4 | 5 |
| b.　Did work or other activities less **carefully** than usual | 1 | 2 | 3 | 4 | 5 |

5. During the **past 4 weeks**, how much did **pain** interfere with your normal work (including both work outside the home and housework)?

| Not at all | A little bit | Moderately | Quite a bit | Extremely |
| --- | --- | --- | --- | --- |
| 1 | 2 | 3 | 4 | 5 |

6. These questions are about how you feel and how things have been with you during the **past 4 weeks**. For each question, please give the one answer that comes closest to the way you have been feeling. How much of the time during the **past 4 weeks**...

|  | All of the time | Most of the time | Some of the time | A little of the time | None of the time |
| --- | --- | --- | --- | --- | --- |
| a. have you felt calm and peaceful? | 1 | 2 | 3 | 4 | 5 |
| b. did you have a lot of energy? | 1 | 2 | 3 | 4 | 5 |
| c. have you felt downhearted and depressed? | 1 | 2 | 3 | 4 | 5 |

7. During the **past 4 weeks**, how much of the time has your **physical health** or **emotional problems** interfered with your social activities (like visiting friends, relatives, etc.)?

| All of the time | Most of the time | Some of the time | A little of the time | None of the time |  |
| --- | --- | --- | --- | --- | --- |
| 1 | 2 | 3 | 4 | 5 | |

**Part I. Knowledge on Hypertension:**

1. **Target blood pressure control should be:**

Higher than 140 / 90mmHg

Not higher than 140 / 90mmHg

Lower than 90 / 60mmHg

Don’t know

1. **If suspected side effects occur after taking anti-hypertensive drugs, what should I do?**

Stop taking the drugs immediately

Switch to previous drugs immediately

Continue to take drugs according to doctor’s prescription; if the side effects continue, then revisit the doctor as soon as possible

Reduce the dose of medication by myself

Don’t know

1. **Which of the following exercise should be avoided by hypertensive patients?**

Sprint

Tai chi

Walking

Stretching exercise

Don’t know

1. **Which of the followings is a healthy lifestyle for hypertensive patients?**

Pursue exciting activities

Smoke, drink, have strong tea and coffee

Have enough sleep and a regular schedule

Don’t know

1. **Which of the following food is rich in salt?**

Fresh pork

Luncheon meat

Steamed fish

Don’t know

**Part II. Lifestyle**:

1. **Do/did you smoke?**

Never

Ex-smoker: I used to smoke ____cigarettes every day, have quitted for ____ year(s) ____ month(s)

Current smoker: I smoke _____ cigarettes every day, for ____ year(s) ____ month(s)

1. **Do/did you drink?**

Never

Ex-drinker: I have stopped drinking completely for ____ year(s) ____ month(s)

Current drinker: Type of alcohol: _________; _______ units / week on average

Current drinker: in the past month, I have drunk in a single occasion more than 5 cans of beer / 5 glasses of dinner wine / 5 shots of liquor for ____ times

1. **Diet habit:**
   1. I take ______ fruits every day on average. (please input number of fruits taken)
   2. I take ______ portions of vegetables every day on average (1 portion equals half a bowl).
   3. I take food like salted meat, preserved meat, salted eggs or preserved bean curd ____ times in a week on average.
2. **Do you exercise regularly?**

No

Yes, _______ times every week, _______ minutes each time;

Type of exercise: _________________

1. **Self blood pressure monitoring:**
   1. How often do you monitor blood pressure?

Never (end of questionnaire)

Regularly, _______ times each month

Irregularly, _______ times each month

- 1. Where do you monitor your blood pressure usually?

At home

In community centres

Other sites, please specify: ________________

- 1. Would you adjust the following interventions by yourself according to your blood pressure readings?

No

Adjust the dose of medication

Adjust diet

Adjust exercise

**Part III: Demographic data:**

| 11. What is your marital status? | 1. Single | | | 2. Married | | | | | | 3. Separated or divorced | | | 4. Widowed | | | | | 5. Refuse to answer | |  |
| --- | --- | --- | --- | --- | --- | --- | --- | --- | --- | --- | --- | --- | --- | --- | --- | --- | --- | --- | --- | --- |
|  |  | | | |  | | |  | | | | | |  | | |  | | |  |
| 12. What is the average monthly income of your household from all sources?  $________________ | 1.  Less than $2,000 | 2.  $2,000  \|  $3,999 | | | | 3.  $4,000  \|  $5,999 | 4.  $6,000  \|  $7,999 | | | | 5.  $8,000  \|  $9,999 | | | | 6.  $10,000  \|  $14,999 | 7.  $15,000  \|  $19,999 | | | 8.  $20,000  \|  $24,999 | |
|  | 9.  $25,000  \|  $29,999 | 10.  $30,000  \|  $39,999 | | | | 11.  $40,000  \|  $59,999 | 12.  $60,000  or  above | | | | 13.  No income | | | | 14.  Refuse to  answer | 15.  Don’t know | | |  | |
|  |  | | | |  | | |  | | | | | |  | | |  | | | |
| 13. What is your average monthly income, including all sources?  $________________ | 1.  Less than $2,000 | | 2.  $2,001  \|  $5,000 | | | | | | 3.  $5,001  \|  $9,999 | | | 4.  $10,000  \|  $14,999 | | | | | | 5.  $15,000  \|  $19,999 | | |
|  | 6.  $20,000  \|  $29,999 | | 7.  $30,000  or  above | | | | | | 8.  No income | | | 9.  Refuse to answer | | | | | | 10.  Don’t know | | |

**The Patient Enablement Instrument**

As a result of your participation in the Risk Assessment Management Programme, do you feel you are ….
(please 🗸 one box in each row):-

|  | Much  Better |  | | | Not  Applicable |
| --- | --- | --- | --- | --- | --- |
|  |  | Better | Same | Less |  |
|  | 2 | 1 | 0 | 0 |  |
| Able to cope with life |  |  |  |  |  |
| Able to understand your illness |  |  |  |  |  |
| Able to cope with your illness |  |  |  |  |  |
| Able to keep yourself healthy |  |  |  |  |  |

|  | Much  Better |  |  |  | Not  Applicable |
| --- | --- | --- | --- | --- | --- |
|  |  | Better | Same | Less |  |
|  | 2 | 1 | 0 | 0 |  |
| Confident about your health |  |  |  |  |  |
| Able to help yourself |  |  |  |  |  |

**Global Rating on Change Scale**

How would you rate the change in your health condition after participating in the Risk Assessment Management Programme?

| **MUCH BETTER** | **BETTER** | **A LITTLE BETTER** | **SAME** | **A LITTLE WORSE** | **WORSE** | **MUCH**  **WORSE** |
| --- | --- | --- | --- | --- | --- | --- |
| +3 | +2 | +1 | 0 | -1 | -2 | -3 |

------------------------------------------------- End ---------------------------------------
